# (20*S*)-20-Acet­oxy-4-pregnene-3,16-dione from *Commiphora wightii*

**DOI:** 10.1107/S1600536810047987

**Published:** 2010-11-27

**Authors:** Sammer Yousuf, Rida Ahmed, Zulfiqar Ali, M. Iqbal Choudhary, Seik Weng Ng

**Affiliations:** a H.E.J. Research Institute of Chemistry, International Center for Chemical and Biological Sciences, University of Karachi, Karachi 75270, Pakistan; b Department of Chemistry, University of Malaya, 50603 Kuala Lumpur, Malaysia

## Abstract

The title triterpene compound, C_23_H_32_O_4_, isolated from *Commiphora wightii* features four *trans*-fused rings, among which the five-membered ring adopts an envelope conformation, the cyclo­hexene ring adopts a half-chair conformation and the two cyclo­hexane rings exist in chair conformations. The asymmetric unit contains two independent mol­ecules. Weak inter­molecular C—H⋯O hydrogen bonding is present in the crystal structure.

## Related literature

For the crystal structures of similar steroids and terpene analogues and other related structures, see: Coiro *et al.* (1982 ▶); Geise *et al.* (1966) ▶. For the isolation of the title compound from other plants, see: Francis *et al.* (2004 ▶); Hung *et al.* (1995 ▶); Zhu *et al.* (2001 ▶).
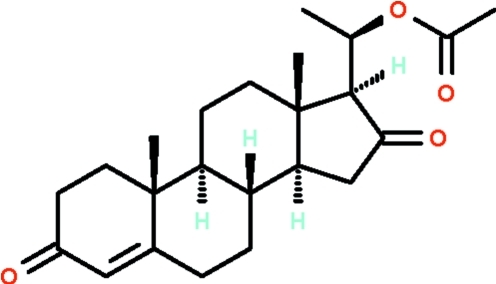


## Experimental

### 

#### Crystal data

C_23_H_32_O_4_
*M* *_r_* = 372.49
Triclinic, 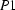

*a* = 8.2802 (4) Å
*b* = 8.6819 (5) Å
*c* = 14.1363 (7) Å
α = 94.130 (1)°
β = 97.028 (1)°
γ = 90.603 (1)°
*V* = 1005.78 (9) Å^3^
*Z* = 2
Mo *K*α radiation
μ = 0.08 mm^−1^
*T* = 293 K
0.4 × 0.3 × 0.2 mm

#### Data collection

Bruker SMART APEX diffractometer
4968 measured reflections
3440 independent reflections
3340 reflections with *I* > 2σ(*I*)
*R*_int_ = 0.028

#### Refinement

*R*[*F* ^2^ > 2σ(*F* ^2^)] = 0.041
*wR*(*F* ^2^) = 0.111
*S* = 1.05
3440 reflections
495 parameters
3 restraints
H-atom parameters constrained
Δρ_max_ = 0.28 e Å^−3^
Δρ_min_ = −0.22 e Å^−3^

### 

Data collection: *SMART* (Bruker, 2002 ▶); cell refinement: *SAINT* (Bruker, 2002 ▶); data reduction: *SAINT*; program(s) used to solve structure: *SHELXS97* (Sheldrick, 2008 ▶); program(s) used to refine structure: *SHELXL97* (Sheldrick, 2008 ▶); molecular graphics: *X-SEED* (Barbour, 2001 ▶); software used to prepare material for publication: *publCIF* (Westrip, 2010 ▶).

## Supplementary Material

Crystal structure: contains datablocks global, I. DOI: 10.1107/S1600536810047987/xu5092sup1.cif

Structure factors: contains datablocks I. DOI: 10.1107/S1600536810047987/xu5092Isup2.hkl

Additional supplementary materials:  crystallographic information; 3D view; checkCIF report

## Figures and Tables

**Table 1:** Hydrogen-bond geometry (Å, °)

*D*—H⋯*A*	*D*—H	H⋯*A*	*D*⋯*A*	*D*—H⋯*A*
C23—H23*B*⋯O2^i^	0.96	2.46	3.393 (4)	165
C46—H46*B*⋯O6^i^	0.96	2.49	3.431 (4)	167

Symmetry code: (i) 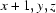.

**Fig. 1.**
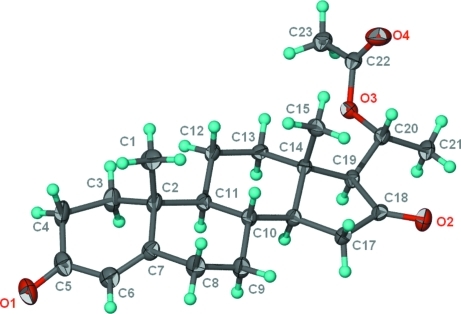
Thermal ellipsoid plot (Barbour, 2001) of one molecule of C23H32O4 at the 50% probability level; hydrogen atoms are drawn as spheres of arbitrary radius.

**Fig. 2.**
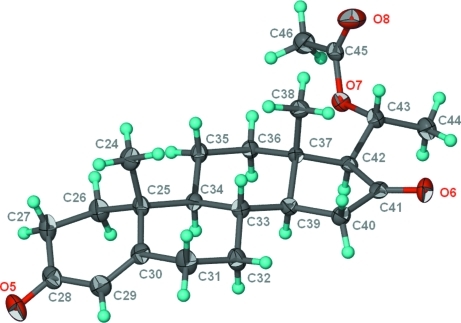
Thermal ellipsoid plot (Barbour, 2001) of second molecule of C23H32O4 at the 50% probability level; hydrogen atoms are drawn as spheres of arbitrary radius.

## supplementary crystallographic information

### Comment

20(*S*)-Acetyloxy-4-pregnene-3,16-dione (Scheme I) has been isolated from the gum resin of *Ailanthus grandis* and *Commiphora mukul* (Francis *et al.*, 2004; Hung *et al.*, 1995). *Commiphora wightii* (Arnott.), a medicinal plant native to parts of Pakistan and India (Zhu *et al.*, 2001), also yields this compound. The triterpene, C_23_H_32_O_4_, features four *trans* fused-rings A–D. Rings B and C exist in a chair conformation whereas ring A adopts a half-chair conformation and ring D an envelope conformation. There are two independent molecules that have similar bond dimensions (Figs. 1 & 2). The stereochemistry is that established in others steroids and terpenes (Coiro *et al.*, 1982; Geise *et al.,* 1966).

### Experimental

Gum resin samples of *Commiphora wightii* were collected from different locations of Pakistan and India, and were deposited at the National Center for Natural Products Research, University of Mississippi. Gum resin (1.5 kg) was extracted with ethyl acetate (5.0 *L* × 72 h) at room temperature; the extract was evaporated to a brown gummy material (250 g). This was subjected to silica-gel column-chromatography and eluted with hexane/ethyl acetate[19:1 (3 *L*), 9:1 (5 *L*), 4:1 (5 *L*), 7:3 (5 *L*), 1:1 (3 *L*), 0:1 (2 *L*)] and acetone (2 *L*) to yield 24 fractions. The seventeenth fraction was subjected to vapor-liquid chromatography on silica followed by elution with methanol/water [3:2 (1.5 *L*), 13:7 (1.5 *L*), 7:3 (1 *L*), 4:1 (2 *L*), 9:1 (3 *L*) and 1:0 (1 *L*)] and acetone (1 *L*)] to give 19 fractions. 20(*S*)-acetyloxy-4-pregnene-3,16-dione (10 mg) was obtained from the sixth fraction and purified by recrystallization from methanol to furnish colorless crystals.

### Refinement

Carbon-bound H-atoms were placed in calculated positions (C–H 0.93 to 0.98 Å) and were included in the refinement in the riding model approximation, with *U*(H) set to 1.2–1.5*U*(C).

### Crystal data

**Table:**

C_23_H_32_O_4_	*Z* = 2
*M**_r_* = 372.49	*F*(000) = 404
Triclinic, *P*1	*D*_x_ = 1.230 Mg m^−^^3^
Hall symbol: P 1	Mo *K*α radiation, λ = 0.71073 Å
*a* = 8.2802 (4) Å	Cell parameters from 3395 reflections
*b* = 8.6819 (5) Å	θ = 2.4–28.3°
*c* = 14.1363 (7) Å	µ = 0.08 mm^−^^1^
α = 94.130 (1)°	*T* = 293 K
β = 97.028 (1)°	Block, colorless
γ = 90.603 (1)°	0.4 × 0.3 × 0.2 mm
*V* = 1005.78 (9) Å^3^	

### Data collection

**Table:**

Bruker SMART APEX diffractometer	3340 reflections with *I* > 2σ(*I*)
Radiation source: fine-focus sealed tube	*R*_int_ = 0.028
graphite	θ_max_ = 25.0°, θ_min_ = 1.5°
ω scans	*h* = −9→9
4968 measured reflections	*k* = −10→9
3440 independent reflections	*l* = −7→16

### Refinement

**Table:**

Refinement on *F*^2^	Primary atom site location: structure-invariant direct methods
Least-squares matrix: full	Secondary atom site location: difference Fourier map
*R*[*F*^2^ > 2σ(*F*^2^)] = 0.041	Hydrogen site location: inferred from neighbouring sites
*wR*(*F*^2^) = 0.111	H-atom parameters constrained
*S* = 1.05	*w* = 1/[σ^2^(*F*_o_^2^) + (0.080*P*)^2^ + 0.1003*P*] where *P* = (*F*_o_^2^ + 2*F*_c_^2^)/3
3440 reflections	(Δ/σ)_max_ = 0.001
495 parameters	Δρ_max_ = 0.28 e Å^−^^3^
3 restraints	Δρ_min_ = −0.22 e Å^−^^3^

### Special details

**Table:**

Geometry. All esds (except the esd in the dihedral angle between two l.s. planes) are estimated using the full covariance matrix. The cell esds are taken into account individually in the estimation of esds in distances, angles and torsion angles; correlations between esds in cell parameters are only used when they are defined by crystal symmetry. An approximate (isotropic) treatment of cell esds is used for estimating esds involving l.s. planes.

### Fractional atomic coordinates and isotropic or equivalent isotropic displacement parameters (Å^2^)

**Table:**

	*x*	*y*	*z*	*U*_iso_*/*U*_eq_	
O1	0.2485 (3)	0.2488 (3)	0.00029 (16)	0.0335 (6)	
O2	−0.3659 (3)	0.3251 (3)	0.65217 (16)	0.0250 (5)	
O3	0.1541 (2)	0.3512 (2)	0.75785 (14)	0.0192 (4)	
O4	0.1920 (3)	0.5699 (3)	0.85416 (18)	0.0335 (6)	
O5	1.0521 (3)	1.0060 (3)	1.23280 (15)	0.0321 (6)	
O6	0.1200 (3)	0.9567 (2)	0.59102 (15)	0.0237 (5)	
O7	0.5908 (2)	0.9323 (2)	0.47823 (14)	0.0189 (4)	
O8	0.5936 (3)	0.7041 (3)	0.39246 (18)	0.0346 (6)	
C1	0.1898 (4)	0.6166 (4)	0.2800 (2)	0.0268 (7)	
H1A	0.2111	0.6509	0.2196	0.040*	
H1B	0.2814	0.6425	0.3272	0.040*	
H1C	0.0947	0.6663	0.2992	0.040*	
C2	0.1611 (4)	0.4405 (3)	0.2706 (2)	0.0200 (6)	
C3	0.3192 (4)	0.3581 (4)	0.2519 (2)	0.0269 (7)	
H3A	0.4079	0.4015	0.2975	0.032*	
H3B	0.3077	0.2496	0.2625	0.032*	
C4	0.3626 (4)	0.3723 (4)	0.1505 (2)	0.0298 (7)	
H4A	0.3864	0.4796	0.1419	0.036*	
H4B	0.4595	0.3136	0.1420	0.036*	
C5	0.2258 (4)	0.3138 (4)	0.0768 (2)	0.0256 (7)	
C6	0.0621 (4)	0.3419 (4)	0.1015 (2)	0.0236 (7)	
H6	−0.0253	0.3167	0.0549	0.028*	
C7	0.0292 (4)	0.4017 (3)	0.1871 (2)	0.0199 (6)	
C8	−0.1438 (4)	0.4368 (4)	0.2032 (2)	0.0252 (7)	
H8A	−0.2171	0.3968	0.1479	0.030*	
H8B	−0.1566	0.5478	0.2104	0.030*	
C9	−0.1890 (4)	0.3649 (4)	0.2923 (2)	0.0233 (7)	
H9A	−0.2987	0.3933	0.3028	0.028*	
H9B	−0.1860	0.2532	0.2830	0.028*	
C10	−0.0702 (3)	0.4211 (3)	0.3791 (2)	0.0172 (6)	
H10	−0.0778	0.5335	0.3895	0.021*	
C11	0.1060 (3)	0.3806 (3)	0.3636 (2)	0.0171 (6)	
H11	0.1089	0.2677	0.3558	0.021*	
C12	0.2261 (4)	0.4297 (3)	0.45342 (19)	0.0183 (6)	
H12A	0.3336	0.3936	0.4435	0.022*	
H12B	0.2318	0.5416	0.4617	0.022*	
C13	0.1786 (3)	0.3666 (3)	0.5454 (2)	0.0174 (6)	
H13A	0.1887	0.2552	0.5416	0.021*	
H13B	0.2527	0.4092	0.5997	0.021*	
C14	0.0042 (3)	0.4081 (3)	0.56053 (19)	0.0150 (6)	
C15	−0.0106 (4)	0.5830 (3)	0.5827 (2)	0.0200 (6)	
H15A	0.0221	0.6368	0.5310	0.030*	
H15B	0.0583	0.6148	0.6406	0.030*	
H15C	−0.1215	0.6064	0.5904	0.030*	
C16	−0.1070 (3)	0.3466 (3)	0.46947 (19)	0.0160 (6)	
H16	−0.0842	0.2365	0.4595	0.019*	
C17	−0.2799 (4)	0.3562 (3)	0.4976 (2)	0.0197 (6)	
H17A	−0.3264	0.4561	0.4857	0.024*	
H17B	−0.3503	0.2760	0.4626	0.024*	
C18	−0.2555 (4)	0.3326 (3)	0.6037 (2)	0.0180 (6)	
C19	−0.0724 (3)	0.3211 (3)	0.6372 (2)	0.0166 (6)	
H19	−0.0441	0.2121	0.6285	0.020*	
C20	−0.0217 (3)	0.3736 (3)	0.7419 (2)	0.0183 (6)	
H20	−0.0455	0.4832	0.7533	0.022*	
C21	−0.0954 (4)	0.2809 (4)	0.8131 (2)	0.0227 (6)	
H21A	−0.0495	0.3160	0.8767	0.034*	
H21B	−0.0724	0.1736	0.8013	0.034*	
H21C	−0.2111	0.2944	0.8063	0.034*	
C22	0.2442 (4)	0.4532 (3)	0.8198 (2)	0.0198 (6)	
C23	0.4153 (4)	0.3996 (4)	0.8376 (2)	0.0249 (7)	
H23A	0.4812	0.4786	0.8755	0.037*	
H23B	0.4572	0.3787	0.7777	0.037*	
H23C	0.4175	0.3072	0.8711	0.037*	
C24	0.8266 (4)	0.6592 (4)	0.9568 (2)	0.0248 (6)	
H24A	0.8741	0.6256	1.0170	0.037*	
H24B	0.8924	0.6262	0.9080	0.037*	
H24C	0.7190	0.6152	0.9410	0.037*	
C25	0.8174 (4)	0.8369 (3)	0.96365 (19)	0.0180 (6)	
C26	0.9907 (4)	0.9082 (4)	0.9777 (2)	0.0239 (7)	
H26A	1.0528	0.8587	0.9306	0.029*	
H26B	0.9841	1.0169	0.9664	0.029*	
C27	1.0807 (4)	0.8922 (4)	1.0769 (2)	0.0268 (7)	
H27A	1.1021	0.7840	1.0850	0.032*	
H27B	1.1846	0.9469	1.0827	0.032*	
C28	0.9851 (4)	0.9551 (4)	1.1542 (2)	0.0240 (7)	
C29	0.8083 (4)	0.9441 (4)	1.1330 (2)	0.0253 (7)	
H29	0.7465	0.9798	1.1804	0.030*	
C30	0.7293 (4)	0.8859 (3)	1.0493 (2)	0.0217 (7)	
C31	0.5485 (4)	0.8600 (4)	1.0375 (2)	0.0276 (7)	
H31A	0.5049	0.9065	1.0933	0.033*	
H31B	0.5246	0.7500	1.0335	0.033*	
C32	0.4645 (4)	0.9291 (4)	0.9476 (2)	0.0256 (7)	
H32A	0.3490	0.9038	0.9400	0.031*	
H32B	0.4773	1.0407	0.9544	0.031*	
C33	0.5384 (4)	0.8652 (3)	0.8596 (2)	0.0180 (6)	
H33	0.5187	0.7533	0.8512	0.022*	
C34	0.7233 (3)	0.8976 (3)	0.87123 (19)	0.0161 (6)	
H34	0.7366	1.0103	0.8785	0.019*	
C35	0.7982 (4)	0.8454 (3)	0.7801 (2)	0.0184 (6)	
H35	0.7948	0.7334	0.7723	0.022*	
H35B	0.9115	0.8787	0.7879	0.022*	
C36	0.7109 (3)	0.9090 (3)	0.6891 (2)	0.0177 (6)	
H36A	0.7282	1.0200	0.6922	0.021*	
H36B	0.7573	0.8642	0.6339	0.021*	
C37	0.5281 (3)	0.8725 (3)	0.67725 (19)	0.0150 (6)	
C38	0.4955 (4)	0.6982 (3)	0.6570 (2)	0.0204 (6)	
H38A	0.5381	0.6634	0.5991	0.031*	
H38B	0.3803	0.6775	0.6499	0.031*	
H38C	0.5475	0.6447	0.7091	0.031*	
C39	0.4629 (3)	0.9379 (3)	0.76972 (19)	0.0167 (6)	
H39	0.4946	1.0476	0.7781	0.020*	
C40	0.2778 (4)	0.9317 (4)	0.7444 (2)	0.0205 (6)	
H40A	0.2329	0.8335	0.7585	0.025*	
H40B	0.2273	1.0144	0.7795	0.025*	
C41	0.2523 (4)	0.9512 (3)	0.6378 (2)	0.0184 (6)	
C42	0.4199 (3)	0.9600 (3)	0.6019 (2)	0.0162 (6)	
H42	0.4559	1.0686	0.6100	0.019*	
C43	0.4216 (4)	0.9065 (3)	0.4966 (2)	0.0192 (6)	
H43	0.3931	0.7962	0.4862	0.023*	
C44	0.3164 (4)	0.9962 (4)	0.4253 (2)	0.0240 (7)	
H44A	0.3299	0.9570	0.3615	0.036*	
H44B	0.3481	1.1035	0.4339	0.036*	
H44C	0.2043	0.9850	0.4352	0.036*	
C45	0.6574 (4)	0.8269 (3)	0.4217 (2)	0.0204 (6)	
C46	0.8215 (4)	0.8812 (4)	0.4021 (2)	0.0262 (7)	
H46A	0.8667	0.8047	0.3609	0.039*	
H46B	0.8922	0.8973	0.4612	0.039*	
H46C	0.8110	0.9765	0.3715	0.039*	

### Atomic displacement parameters (Å^2^)

**Table:**

	*U*^11^	*U*^22^	*U*^33^	*U*^12^	*U*^13^	*U*^23^
O1	0.0412 (15)	0.0379 (13)	0.0235 (12)	0.0034 (11)	0.0133 (10)	0.0012 (10)
O2	0.0210 (11)	0.0307 (12)	0.0252 (11)	−0.0001 (9)	0.0088 (9)	0.0052 (9)
O3	0.0203 (11)	0.0211 (10)	0.0161 (10)	−0.0008 (9)	0.0024 (8)	0.0001 (8)
O4	0.0271 (12)	0.0255 (12)	0.0452 (14)	−0.0027 (10)	0.0017 (10)	−0.0113 (10)
O5	0.0362 (14)	0.0370 (13)	0.0204 (11)	−0.0050 (11)	−0.0074 (10)	0.0026 (10)
O6	0.0159 (11)	0.0266 (11)	0.0278 (11)	−0.0007 (9)	−0.0031 (9)	0.0060 (9)
O7	0.0198 (11)	0.0203 (10)	0.0167 (10)	−0.0015 (8)	0.0027 (8)	0.0023 (8)
O8	0.0313 (14)	0.0257 (12)	0.0463 (15)	−0.0016 (10)	0.0102 (11)	−0.0094 (10)
C1	0.0327 (18)	0.0287 (16)	0.0193 (15)	−0.0077 (13)	0.0014 (13)	0.0081 (12)
C2	0.0175 (15)	0.0272 (16)	0.0162 (14)	−0.0026 (12)	0.0041 (11)	0.0062 (12)
C3	0.0210 (16)	0.0415 (19)	0.0195 (15)	0.0023 (14)	0.0048 (12)	0.0062 (13)
C4	0.0222 (17)	0.046 (2)	0.0244 (16)	0.0004 (14)	0.0113 (14)	0.0072 (14)
C5	0.0350 (19)	0.0254 (16)	0.0187 (15)	0.0040 (14)	0.0087 (13)	0.0078 (12)
C6	0.0259 (17)	0.0267 (16)	0.0184 (14)	−0.0007 (13)	0.0009 (12)	0.0070 (12)
C7	0.0199 (16)	0.0214 (15)	0.0191 (14)	−0.0005 (12)	0.0025 (12)	0.0065 (11)
C8	0.0218 (16)	0.0356 (17)	0.0182 (15)	0.0033 (13)	0.0003 (12)	0.0057 (13)
C9	0.0181 (15)	0.0329 (17)	0.0193 (15)	0.0006 (13)	0.0032 (12)	0.0025 (12)
C10	0.0176 (15)	0.0192 (14)	0.0155 (14)	0.0001 (11)	0.0038 (11)	0.0027 (11)
C11	0.0173 (15)	0.0202 (14)	0.0143 (13)	−0.0006 (11)	0.0028 (11)	0.0030 (11)
C12	0.0153 (14)	0.0237 (14)	0.0165 (14)	−0.0036 (12)	0.0033 (11)	0.0031 (11)
C13	0.0163 (15)	0.0203 (14)	0.0153 (13)	−0.0046 (12)	0.0003 (11)	0.0018 (11)
C14	0.0167 (14)	0.0154 (13)	0.0133 (13)	−0.0024 (11)	0.0032 (11)	0.0025 (10)
C15	0.0270 (16)	0.0187 (14)	0.0147 (13)	−0.0030 (12)	0.0033 (11)	0.0021 (11)
C16	0.0161 (14)	0.0177 (14)	0.0143 (13)	0.0005 (11)	0.0019 (11)	0.0031 (11)
C17	0.0153 (14)	0.0221 (14)	0.0222 (15)	−0.0014 (11)	0.0043 (12)	0.0012 (12)
C18	0.0181 (15)	0.0128 (13)	0.0239 (14)	−0.0022 (11)	0.0068 (12)	−0.0009 (11)
C19	0.0191 (15)	0.0137 (13)	0.0183 (14)	0.0008 (11)	0.0066 (11)	0.0032 (10)
C20	0.0182 (15)	0.0182 (14)	0.0193 (14)	−0.0017 (12)	0.0050 (11)	0.0030 (11)
C21	0.0239 (16)	0.0269 (16)	0.0184 (14)	−0.0044 (13)	0.0065 (12)	0.0042 (12)
C22	0.0225 (16)	0.0197 (15)	0.0179 (14)	−0.0049 (12)	0.0047 (12)	0.0025 (12)
C23	0.0252 (17)	0.0266 (16)	0.0233 (15)	−0.0054 (13)	0.0038 (13)	0.0048 (12)
C24	0.0292 (17)	0.0247 (15)	0.0208 (14)	0.0026 (13)	0.0005 (12)	0.0072 (12)
C25	0.0185 (14)	0.0234 (15)	0.0125 (13)	0.0005 (12)	0.0010 (11)	0.0050 (11)
C26	0.0223 (16)	0.0302 (16)	0.0197 (15)	−0.0018 (13)	0.0035 (12)	0.0044 (12)
C27	0.0184 (16)	0.0375 (18)	0.0242 (16)	−0.0020 (14)	−0.0020 (12)	0.0094 (13)
C28	0.0307 (17)	0.0233 (15)	0.0176 (15)	−0.0009 (13)	−0.0019 (13)	0.0064 (12)
C29	0.0321 (18)	0.0293 (16)	0.0155 (14)	0.0049 (14)	0.0040 (13)	0.0048 (12)
C30	0.0254 (17)	0.0220 (15)	0.0184 (14)	0.0035 (13)	0.0021 (12)	0.0069 (12)
C31	0.0216 (16)	0.046 (2)	0.0167 (15)	−0.0016 (14)	0.0069 (12)	0.0046 (13)
C32	0.0175 (15)	0.0396 (19)	0.0194 (15)	0.0011 (14)	0.0025 (12)	−0.0003 (13)
C33	0.0170 (15)	0.0198 (14)	0.0175 (14)	−0.0008 (11)	0.0025 (11)	0.0028 (11)
C34	0.0176 (15)	0.0153 (13)	0.0156 (14)	0.0011 (11)	0.0016 (11)	0.0029 (11)
C35	0.0137 (14)	0.0229 (15)	0.0189 (14)	−0.0002 (11)	0.0022 (11)	0.0026 (11)
C36	0.0153 (14)	0.0224 (14)	0.0154 (13)	0.0012 (11)	0.0024 (11)	0.0006 (11)
C37	0.0135 (14)	0.0169 (14)	0.0143 (13)	0.0014 (11)	0.0006 (11)	−0.0003 (10)
C38	0.0242 (15)	0.0178 (14)	0.0191 (14)	0.0020 (12)	0.0020 (12)	0.0011 (11)
C39	0.0160 (14)	0.0183 (14)	0.0160 (14)	−0.0009 (11)	0.0031 (11)	0.0007 (11)
C40	0.0160 (15)	0.0233 (15)	0.0224 (15)	0.0005 (11)	0.0041 (11)	0.0004 (12)
C41	0.0170 (15)	0.0111 (13)	0.0262 (15)	−0.0021 (11)	0.0010 (12)	−0.0015 (11)
C42	0.0196 (15)	0.0125 (13)	0.0160 (13)	−0.0021 (11)	0.0005 (11)	−0.0005 (10)
C43	0.0185 (15)	0.0225 (15)	0.0160 (14)	−0.0010 (12)	0.0003 (11)	0.0013 (11)
C44	0.0261 (16)	0.0276 (16)	0.0181 (15)	−0.0001 (13)	−0.0001 (12)	0.0040 (12)
C45	0.0257 (16)	0.0208 (15)	0.0137 (13)	0.0033 (12)	−0.0024 (12)	0.0019 (11)
C46	0.0274 (17)	0.0286 (16)	0.0232 (16)	0.0031 (13)	0.0042 (13)	0.0033 (12)

### Geometric parameters (Å, °)

**Table:**

O1—C5	1.220 (4)	C21—H21C	0.9600
O2—C18	1.211 (4)	C22—C23	1.494 (4)
O3—C22	1.349 (4)	C23—H23A	0.9600
O3—C20	1.463 (3)	C23—H23B	0.9600
O4—C22	1.200 (4)	C23—H23C	0.9600
O5—C28	1.229 (4)	C24—C25	1.542 (4)
O6—C41	1.212 (4)	C24—H24A	0.9600
O7—C45	1.339 (4)	C24—H24B	0.9600
O7—C43	1.473 (4)	C24—H24C	0.9600
O8—C45	1.207 (4)	C25—C30	1.526 (4)
C1—C2	1.539 (4)	C25—C26	1.541 (4)
C1—H1A	0.9600	C25—C34	1.566 (4)
C1—H1B	0.9600	C26—C27	1.522 (4)
C1—H1C	0.9600	C26—H26A	0.9700
C2—C7	1.522 (4)	C26—H26B	0.9700
C2—C3	1.540 (4)	C27—C28	1.502 (4)
C2—C11	1.565 (4)	C27—H27A	0.9700
C3—C4	1.532 (4)	C27—H27B	0.9700
C3—H3A	0.9700	C28—C29	1.458 (5)
C3—H3B	0.9700	C29—C30	1.341 (4)
C4—C5	1.500 (5)	C29—H29	0.9300
C4—H4A	0.9700	C30—C31	1.499 (4)
C4—H4B	0.9700	C31—C32	1.536 (4)
C5—C6	1.459 (4)	C31—H31A	0.9700
C6—C7	1.342 (4)	C31—H31B	0.9700
C6—H6	0.9300	C32—C33	1.525 (4)
C7—C8	1.509 (4)	C32—H32A	0.9700
C8—C9	1.529 (4)	C32—H32B	0.9700
C8—H8A	0.9700	C33—C39	1.528 (4)
C8—H8B	0.9700	C33—C34	1.540 (4)
C9—C10	1.523 (4)	C33—H33	0.9800
C9—H9A	0.9700	C34—C35	1.538 (4)
C9—H9B	0.9700	C34—H34	0.9800
C10—C16	1.534 (4)	C35—C36	1.538 (4)
C10—C11	1.542 (4)	C35—H35	0.9700
C10—H10	0.9800	C35—H35B	0.9700
C11—C12	1.545 (4)	C36—C37	1.530 (4)
C11—H11	0.9800	C36—H36A	0.9700
C12—C13	1.539 (4)	C36—H36B	0.9700
C12—H12A	0.9700	C37—C38	1.534 (4)
C12—H12B	0.9700	C37—C39	1.548 (4)
C13—C14	1.528 (4)	C37—C42	1.553 (4)
C13—H13A	0.9700	C38—H38A	0.9600
C13—H13B	0.9700	C38—H38B	0.9600
C14—C15	1.537 (4)	C38—H38C	0.9600
C14—C16	1.546 (4)	C39—C40	1.530 (4)
C14—C19	1.558 (4)	C39—H39	0.9800
C15—H15A	0.9600	C40—C41	1.518 (4)
C15—H15B	0.9600	C40—H40A	0.9700
C15—H15C	0.9600	C40—H40B	0.9700
C16—C17	1.533 (4)	C41—C42	1.538 (4)
C16—H16	0.9800	C42—C43	1.529 (4)
C17—C18	1.518 (4)	C42—H42	0.9800
C17—H17A	0.9700	C43—C44	1.515 (4)
C17—H17B	0.9700	C43—H43	0.9800
C18—C19	1.538 (4)	C44—H44A	0.9600
C19—C20	1.523 (4)	C44—H44B	0.9600
C19—H19	0.9800	C44—H44C	0.9600
C20—C21	1.512 (4)	C45—C46	1.498 (4)
C20—H20	0.9800	C46—H46A	0.9600
C21—H21A	0.9600	C46—H46B	0.9600
C21—H21B	0.9600	C46—H46C	0.9600
			
C22—O3—C20	117.8 (2)	H23B—C23—H23C	109.5
C45—O7—C43	118.4 (2)	C25—C24—H24A	109.5
C2—C1—H1A	109.5	C25—C24—H24B	109.5
C2—C1—H1B	109.5	H24A—C24—H24B	109.5
H1A—C1—H1B	109.5	C25—C24—H24C	109.5
C2—C1—H1C	109.5	H24A—C24—H24C	109.5
H1A—C1—H1C	109.5	H24B—C24—H24C	109.5
H1B—C1—H1C	109.5	C30—C25—C26	109.9 (2)
C7—C2—C1	108.1 (2)	C30—C25—C24	107.9 (2)
C7—C2—C3	109.8 (2)	C26—C25—C24	109.7 (2)
C1—C2—C3	110.1 (3)	C30—C25—C34	109.3 (2)
C7—C2—C11	109.3 (2)	C26—C25—C34	108.2 (2)
C1—C2—C11	111.7 (2)	C24—C25—C34	111.8 (2)
C3—C2—C11	107.8 (2)	C27—C26—C25	113.5 (2)
C4—C3—C2	113.3 (3)	C27—C26—H26A	108.9
C4—C3—H3A	108.9	C25—C26—H26A	108.9
C2—C3—H3A	108.9	C27—C26—H26B	108.9
C4—C3—H3B	108.9	C25—C26—H26B	108.9
C2—C3—H3B	108.9	H26A—C26—H26B	107.7
H3A—C3—H3B	107.7	C28—C27—C26	111.9 (3)
C5—C4—C3	111.2 (3)	C28—C27—H27A	109.2
C5—C4—H4A	109.4	C26—C27—H27A	109.2
C3—C4—H4A	109.4	C28—C27—H27B	109.2
C5—C4—H4B	109.4	C26—C27—H27B	109.2
C3—C4—H4B	109.4	H27A—C27—H27B	107.9
H4A—C4—H4B	108.0	O5—C28—C29	121.8 (3)
O1—C5—C6	121.6 (3)	O5—C28—C27	121.7 (3)
O1—C5—C4	122.6 (3)	C29—C28—C27	116.4 (3)
C6—C5—C4	115.7 (3)	C30—C29—C28	124.1 (3)
C7—C6—C5	124.3 (3)	C30—C29—H29	117.9
C7—C6—H6	117.8	C28—C29—H29	117.9
C5—C6—H6	117.8	C29—C30—C31	120.7 (3)
C6—C7—C8	120.1 (3)	C29—C30—C25	122.5 (3)
C6—C7—C2	122.7 (3)	C31—C30—C25	116.8 (3)
C8—C7—C2	117.1 (3)	C30—C31—C32	112.1 (3)
C7—C8—C9	111.3 (2)	C30—C31—H31A	109.2
C7—C8—H8A	109.4	C32—C31—H31A	109.2
C9—C8—H8A	109.4	C30—C31—H31B	109.2
C7—C8—H8B	109.4	C32—C31—H31B	109.2
C9—C8—H8B	109.4	H31A—C31—H31B	107.9
H8A—C8—H8B	108.0	C33—C32—C31	110.1 (3)
C10—C9—C8	110.0 (3)	C33—C32—H32A	109.6
C10—C9—H9A	109.7	C31—C32—H32A	109.6
C8—C9—H9A	109.7	C33—C32—H32B	109.6
C10—C9—H9B	109.7	C31—C32—H32B	109.6
C8—C9—H9B	109.7	H32A—C32—H32B	108.1
H9A—C9—H9B	108.2	C32—C33—C39	111.3 (2)
C9—C10—C16	111.7 (2)	C32—C33—C34	110.9 (2)
C9—C10—C11	110.9 (2)	C39—C33—C34	108.0 (2)
C16—C10—C11	107.3 (2)	C32—C33—H33	108.9
C9—C10—H10	109.0	C39—C33—H33	108.9
C16—C10—H10	109.0	C34—C33—H33	108.9
C11—C10—H10	109.0	C35—C34—C33	111.7 (2)
C10—C11—C12	111.1 (2)	C35—C34—C25	112.8 (2)
C10—C11—C2	114.1 (2)	C33—C34—C25	114.1 (2)
C12—C11—C2	112.4 (2)	C35—C34—H34	105.8
C10—C11—H11	106.2	C33—C34—H34	105.8
C12—C11—H11	106.2	C25—C34—H34	105.8
C2—C11—H11	106.2	C34—C35—C36	113.5 (2)
C13—C12—C11	113.5 (2)	C34—C35—H35	108.9
C13—C12—H12A	108.9	C36—C35—H35	108.9
C11—C12—H12A	108.9	C34—C35—H35B	108.9
C13—C12—H12B	108.9	C36—C35—H35B	108.9
C11—C12—H12B	108.9	H35—C35—H35B	107.7
H12A—C12—H12B	107.7	C37—C36—C35	111.6 (2)
C14—C13—C12	111.3 (2)	C37—C36—H36A	109.3
C14—C13—H13A	109.4	C35—C36—H36A	109.3
C12—C13—H13A	109.4	C37—C36—H36B	109.3
C14—C13—H13B	109.4	C35—C36—H36B	109.3
C12—C13—H13B	109.4	H36A—C36—H36B	108.0
H13A—C13—H13B	108.0	C36—C37—C38	110.7 (2)
C13—C14—C15	111.0 (2)	C36—C37—C39	107.0 (2)
C13—C14—C16	107.1 (2)	C38—C37—C39	112.7 (2)
C15—C14—C16	112.7 (2)	C36—C37—C42	116.8 (2)
C13—C14—C19	116.4 (2)	C38—C37—C42	109.1 (2)
C15—C14—C19	109.1 (2)	C39—C37—C42	100.1 (2)
C16—C14—C19	100.1 (2)	C37—C38—H38A	109.5
C14—C15—H15A	109.5	C37—C38—H38B	109.5
C14—C15—H15B	109.5	H38A—C38—H38B	109.5
H15A—C15—H15B	109.5	C37—C38—H38C	109.5
C14—C15—H15C	109.5	H38A—C38—H38C	109.5
H15A—C15—H15C	109.5	H38B—C38—H38C	109.5
H15B—C15—H15C	109.5	C33—C39—C40	119.0 (2)
C17—C16—C10	118.7 (2)	C33—C39—C37	113.6 (2)
C17—C16—C14	104.6 (2)	C40—C39—C37	104.2 (2)
C10—C16—C14	113.4 (2)	C33—C39—H39	106.4
C17—C16—H16	106.4	C40—C39—H39	106.4
C10—C16—H16	106.4	C37—C39—H39	106.4
C14—C16—H16	106.4	C41—C40—C39	104.0 (2)
C18—C17—C16	103.3 (2)	C41—C40—H40A	111.0
C18—C17—H17A	111.1	C39—C40—H40A	111.0
C16—C17—H17A	111.1	C41—C40—H40B	111.0
C18—C17—H17B	111.1	C39—C40—H40B	111.0
C16—C17—H17B	111.1	H40A—C40—H40B	109.0
H17A—C17—H17B	109.1	O6—C41—C40	124.1 (3)
O2—C18—C17	123.8 (3)	O6—C41—C42	127.3 (3)
O2—C18—C19	127.0 (3)	C40—C41—C42	108.5 (2)
C17—C18—C19	109.2 (2)	C43—C42—C41	115.0 (2)
C20—C19—C18	114.8 (2)	C43—C42—C37	117.6 (2)
C20—C19—C14	117.7 (2)	C41—C42—C37	102.0 (2)
C18—C19—C14	101.8 (2)	C43—C42—H42	107.2
C20—C19—H19	107.3	C41—C42—H42	107.2
C18—C19—H19	107.3	C37—C42—H42	107.2
C14—C19—H19	107.3	O7—C43—C44	106.1 (2)
O3—C20—C21	106.5 (2)	O7—C43—C42	104.9 (2)
O3—C20—C19	105.3 (2)	C44—C43—C42	116.0 (2)
C21—C20—C19	115.3 (2)	O7—C43—H43	109.9
O3—C20—H20	109.8	C44—C43—H43	109.9
C21—C20—H20	109.8	C42—C43—H43	109.9
C19—C20—H20	109.8	C43—C44—H44A	109.5
C20—C21—H21A	109.5	C43—C44—H44B	109.5
C20—C21—H21B	109.5	H44A—C44—H44B	109.5
H21A—C21—H21B	109.5	C43—C44—H44C	109.5
C20—C21—H21C	109.5	H44A—C44—H44C	109.5
H21A—C21—H21C	109.5	H44B—C44—H44C	109.5
H21B—C21—H21C	109.5	O8—C45—O7	123.9 (3)
O4—C22—O3	124.0 (3)	O8—C45—C46	125.3 (3)
O4—C22—C23	125.9 (3)	O7—C45—C46	110.8 (2)
O3—C22—C23	110.2 (3)	C45—C46—H46A	109.5
C22—C23—H23A	109.5	C45—C46—H46B	109.5
C22—C23—H23B	109.5	H46A—C46—H46B	109.5
H23A—C23—H23B	109.5	C45—C46—H46C	109.5
C22—C23—H23C	109.5	H46A—C46—H46C	109.5
H23A—C23—H23C	109.5	H46B—C46—H46C	109.5
			
C7—C2—C3—C4	45.7 (4)	C30—C25—C26—C27	46.1 (3)
C1—C2—C3—C4	−73.2 (3)	C24—C25—C26—C27	−72.4 (3)
C11—C2—C3—C4	164.8 (3)	C34—C25—C26—C27	165.3 (3)
C2—C3—C4—C5	−55.9 (4)	C25—C26—C27—C28	−54.0 (4)
C3—C4—C5—O1	−146.1 (3)	C26—C27—C28—O5	−152.6 (3)
C3—C4—C5—C6	35.0 (4)	C26—C27—C28—C29	30.9 (4)
O1—C5—C6—C7	174.6 (3)	O5—C28—C29—C30	−178.2 (3)
C4—C5—C6—C7	−6.5 (4)	C27—C28—C29—C30	−1.7 (4)
C5—C6—C7—C8	175.6 (3)	C28—C29—C30—C31	172.1 (3)
C5—C6—C7—C2	−3.0 (5)	C28—C29—C30—C25	−5.4 (5)
C1—C2—C7—C6	103.3 (3)	C26—C25—C30—C29	−17.0 (4)
C3—C2—C7—C6	−16.9 (4)	C24—C25—C30—C29	102.6 (3)
C11—C2—C7—C6	−135.0 (3)	C34—C25—C30—C29	−135.6 (3)
C1—C2—C7—C8	−75.4 (3)	C26—C25—C30—C31	165.4 (3)
C3—C2—C7—C8	164.5 (3)	C24—C25—C30—C31	−75.0 (3)
C11—C2—C7—C8	46.4 (3)	C34—C25—C30—C31	46.8 (3)
C6—C7—C8—C9	128.6 (3)	C29—C30—C31—C32	130.3 (3)
C2—C7—C8—C9	−52.7 (4)	C25—C30—C31—C32	−52.1 (4)
C7—C8—C9—C10	56.5 (3)	C30—C31—C32—C33	55.3 (4)
C8—C9—C10—C16	−177.8 (2)	C31—C32—C33—C39	−177.2 (2)
C8—C9—C10—C11	−58.1 (3)	C31—C32—C33—C34	−56.9 (3)
C9—C10—C11—C12	−177.0 (2)	C32—C33—C34—C35	−175.6 (2)
C16—C10—C11—C12	−54.8 (3)	C39—C33—C34—C35	−53.4 (3)
C9—C10—C11—C2	54.6 (3)	C32—C33—C34—C25	55.0 (3)
C16—C10—C11—C2	176.9 (2)	C39—C33—C34—C25	177.1 (2)
C7—C2—C11—C10	−46.7 (3)	C30—C25—C34—C35	−176.6 (2)
C1—C2—C11—C10	72.9 (3)	C26—C25—C34—C35	63.7 (3)
C3—C2—C11—C10	−166.0 (2)	C24—C25—C34—C35	−57.2 (3)
C7—C2—C11—C12	−174.4 (2)	C30—C25—C34—C33	−47.8 (3)
C1—C2—C11—C12	−54.8 (3)	C26—C25—C34—C33	−167.4 (2)
C3—C2—C11—C12	66.3 (3)	C24—C25—C34—C33	71.6 (3)
C10—C11—C12—C13	53.1 (3)	C33—C34—C35—C36	52.0 (3)
C2—C11—C12—C13	−177.6 (2)	C25—C34—C35—C36	−177.9 (2)
C11—C12—C13—C14	−53.8 (3)	C34—C35—C36—C37	−53.6 (3)
C12—C13—C14—C15	−67.9 (3)	C35—C36—C37—C38	−67.5 (3)
C12—C13—C14—C16	55.5 (3)	C35—C36—C37—C39	55.6 (3)
C12—C13—C14—C19	166.6 (2)	C35—C36—C37—C42	166.8 (2)
C9—C10—C16—C17	−53.0 (3)	C32—C33—C39—C40	−54.3 (3)
C11—C10—C16—C17	−174.8 (2)	C34—C33—C39—C40	−176.2 (2)
C9—C10—C16—C14	−176.4 (2)	C32—C33—C39—C37	−177.6 (2)
C11—C10—C16—C14	61.8 (3)	C34—C33—C39—C37	60.5 (3)
C13—C14—C16—C17	166.9 (2)	C36—C37—C39—C33	−61.6 (3)
C15—C14—C16—C17	−70.7 (3)	C38—C37—C39—C33	60.3 (3)
C19—C14—C16—C17	45.0 (3)	C42—C37—C39—C33	176.1 (2)
C13—C14—C16—C10	−62.3 (3)	C36—C37—C39—C40	167.3 (2)
C15—C14—C16—C10	60.1 (3)	C38—C37—C39—C40	−70.8 (3)
C19—C14—C16—C10	175.9 (2)	C42—C37—C39—C40	45.0 (3)
C10—C16—C17—C18	−158.1 (2)	C33—C39—C40—C41	−157.7 (2)
C14—C16—C17—C18	−30.4 (3)	C37—C39—C40—C41	−29.9 (3)
C16—C17—C18—O2	−176.4 (3)	C39—C40—C41—O6	−177.9 (3)
C16—C17—C18—C19	3.8 (3)	C39—C40—C41—C42	2.8 (3)
O2—C18—C19—C20	−27.7 (4)	O6—C41—C42—C43	−26.0 (4)
C17—C18—C19—C20	152.0 (2)	C40—C41—C42—C43	153.2 (2)
O2—C18—C19—C14	−156.1 (3)	O6—C41—C42—C37	−154.3 (3)
C17—C18—C19—C14	23.7 (3)	C40—C41—C42—C37	24.9 (3)
C13—C14—C19—C20	77.6 (3)	C36—C37—C42—C43	76.4 (3)
C15—C14—C19—C20	−48.9 (3)	C38—C37—C42—C43	−50.1 (3)
C16—C14—C19—C20	−167.4 (2)	C39—C37—C42—C43	−168.6 (2)
C13—C14—C19—C18	−156.0 (2)	C36—C37—C42—C41	−156.9 (2)
C15—C14—C19—C18	77.5 (3)	C38—C37—C42—C41	76.6 (3)
C16—C14—C19—C18	−41.0 (2)	C39—C37—C42—C41	−41.9 (2)
C22—O3—C20—C21	−91.5 (3)	C45—O7—C43—C44	−95.8 (3)
C22—O3—C20—C19	145.7 (2)	C45—O7—C43—C42	141.0 (2)
C18—C19—C20—O3	179.4 (2)	C41—C42—C43—O7	177.4 (2)
C14—C19—C20—O3	−60.8 (3)	C37—C42—C43—O7	−62.5 (3)
C18—C19—C20—C21	62.3 (3)	C41—C42—C43—C44	60.7 (3)
C14—C19—C20—C21	−177.8 (2)	C37—C42—C43—C44	−179.2 (3)
C20—O3—C22—O4	−8.6 (4)	C43—O7—C45—O8	−7.9 (4)
C20—O3—C22—C23	171.8 (2)	C43—O7—C45—C46	173.4 (2)

### Hydrogen-bond geometry (Å, °)

**Table:**

*D*—H···*A*	*D*—H	H···*A*	*D*···*A*	*D*—H···*A*
C23—H23B···O2^i^	0.96	2.46	3.393 (4)	165
C46—H46B···O6^i^	0.96	2.49	3.431 (4)	167

Symmetry codes: (i) *x*+1, *y*, *z*.
